# Cold Cas: reevaluating the occurrence of CRISPR/Cas systems in *Mycobacteriaceae*

**DOI:** 10.3389/fmicb.2023.1204838

**Published:** 2023-06-27

**Authors:** Evan Brenner, Srinand Sreevatsan

**Affiliations:** Department of Pathobiology and Diagnostic Investigation, College of Veterinary Medicine, Michigan State University, East Lansing, MI, United States

**Keywords:** CRISPR, Cas, mycobacteria, non-tuberculous mycobacteria, MAC, *Mycobacterium tuberculosis* complex

## Abstract

Bacterial CRISPR/Cas systems target foreign genetic elements such as phages and regulate gene expression by some pathogens, even in the host. The system is a marker for evolutionary history and has been used for inferences in *Mycobacterium tuberculosis* for 30 years. However, knowledge about mycobacterial CRISPR/Cas systems remains limited. It is believed that Type III-A Cas systems are exclusive to *Mycobacterium canettii* and the *M. tuberculosis* complex (MTBC) of organisms and that very few of the >200 diverse species of non-tuberculous mycobacteria (NTM) possess any CRISPR/Cas system. This study sought unreported CRISPR/Cas loci across NTM to better understand mycobacterial evolution, particularly in species phylogenetically near the MTBC. An analysis of available mycobacterial genomes revealed that Cas systems are widespread across *Mycobacteriaceae* and that some species contain multiple types. The phylogeny of Cas loci shows scattered presence in many NTM, with variation even within species, suggesting gains/losses of these loci occur frequently. Cas Type III-A systems were identified in pathogenic *Mycobacterium heckeshornense* and the geological environmental isolate *Mycobacterium* SM1. In summary, mycobacterial CRISPR/Cas systems are numerous, Type III-A systems are unreliable as markers for MTBC evolution, and mycobacterial horizontal gene transfer appears to be a frequent source of genetic variation.

## Introduction

The impacts of mycobacteria on human and animal health are difficult to overstate. Apart from the well-known effects of the members of the *Mycobacterium tuberculosis* complex (MTBC), *Mycobacterium avium* complex members cause widespread losses in animal agriculture, numerous non-tuberculous mycobacteria cause disease in humans, mycobacterial biofilms pose challenges for water treatment and hospital infection control, and some species are merely innocuous saprophytes. With such diversity, it is perhaps surprising that mycobacterial genomes share much in common. Between *Mycolicibacterium smegmatis*, a distant and non-pathogenic saprophyte, and *M. tuberculosis* (MTB), *M. avium, Mycolicibacterium leprae*, and *Mycolicibacterium abscessus—*four highly divergent species in the *Mycobacterium* genus—there are over 1,000 genes with more than 50% amino acid identity (Judd et al., 2021). While this is less than a quarter of genes in MTB, *M. leprae* has undergone extensive genome reduction and has only 1,600 genes in total (Vissa and Brennan, 2001), meaning this conserved core gene set accounts for ~70% of the *leprae* genome. The closeness of the genetic background between mycobacteria has been discussed since before Illumina sequencing became prevalent and whole genome sequences were rare (Marri et al., 2006), with one argument for this closeness being an apparent dearth of horizontal gene transfer, at least as seen in MTBC organisms (Sreevatsan et al., 1997; Derbyshire and Gray, 2014). In the last 15 years, advances in mycobacterial genomics have revealed horizontal gene transfer occurring through unusual “distributive conjugative transfer” (DCT) mechanisms in *M. smegmatis* (Derbyshire and Gray, 2014; Gray and Derbyshire, 2018). While the extent of the effects of this process on mycobacterial diversity remains debated, it is now generally accepted that mycobacterial species undergo an atypical chromosomal genetic transfer that results in meiosis-like genome mosaics (Veyrier et al., 2009; Boritsch et al., 2014, 2016; Derbyshire and Gray, 2014; Madacki et al., 2021). It remains contested how much, if any, transfer occurs in the MTBC. However, DCT has been demonstrated to occur at low frequencies in *Mycobacterium canettii*, the “smooth tubercule” species at the periphery of the MTBC, and is presumed to be most like the proposed *Mycobacterium prototuberculosis* ancestor of modern MTB (Boritsch et al., 2014, 2016; Singh et al., 2021). The development of some mycobacteria into some of the most resilient and deadly human pathogens has followed an unclear evolutionary trajectory, and our understanding of genes and pathways in these organisms, how they function, and what roles they may or may not play in the pathogenesis or host adaptation is limited. Determining the relatedness of organisms has long been crucial in outbreak investigations. In the 1990s, tuberculosis outbreaks began being differentiated through restriction fragment length polymorphism analysis of mycobacterial insertion sequences (Otal et al., 1991) and later through “spoligotyping,” a technique that exploited unusual loci of spacers and was repeated in the MTB genome (Groenen et al., 1993; Kamerbeek et al., 1997). Subsequently, this pattern of repeats and spacers identified across different bacterial and archaeal genomes became the basis of a genetic revolution as the CRISPR/Cas system began to be understood and exploited for eukaryotic genome engineering (Barrangou et al., 2007; Garneau et al., 2010). While a tremendous amount of research has gone into the study of CRISPR/Cas systems across prokaryotes, the study of the system in mycobacteria has remained fairly limited. It has been speculated that the MTBC CRISPR/Cas system may be non-functional (He et al., 2012) and that CRISPR/Cas loci exist exclusively in the MTBC and *M. canettii* (Supply et al., 2013). However, research by Wei et al. (2019) and Grüschow et al. (2019) reported that the Type III-A system found in the MTBC is unique from other Type III-A systems, is actively expressed, and targets foreign genetic elements through an unusual cyclic hexa-adenylate signaling pathway instead of the expected nuclease-driven DNA cleavage (Grüschow et al., 2019; Wei et al., 2019). More attention has since been paid to *M. canettii* and its diversity, including the multiple CRISPR/Cas system types contained across its strains: Type III-A in STB-A and STB-D, Type I-C in STB-K, STB-L, and STB-J, Type I-E in STB-G, and STB-I and a variant Type I-C in STB-E and STB-H (Supply et al., 2013). The diversity in these systems and their presumed exclusive presence in these species spurred hypotheses that *M. canettii* acquired these systems from other non-mycobacterial environmental species and that an evolutionary history of the MTBC might be able to be derived through the tracing of this uptake (Singh et al., 2021). In 2021, Singh et al. explored 141 *Mycobacteriaceae* genomes to determine if CRISPR/Cas systems existed outside the *M. canettii*/MTBC cluster (Singh et al., 2021). They confirmed that Type III-A systems were exclusive to the *M. canettii*/MTBC group and also identified the rare presence of alternative systems in a handful of other *Mycobacteriaceae*. In this study, searches for CRISPR/Cas loci outside the MTBC revealed a substantial number of previously unannotated CRISPR/Cas systems across *Mycobacteriaceae*. Surprisingly, these systems show very little conservation, with variation in the presence and types of systems even between different isolates of the same species. This distribution of systems suggests that CRISPR/Cas loci regularly undergo exchange through DCT or other means of horizontal gene transfer. Further, with the identification of a Type III-A Cas system in the environmental isolate *Mycobacterium* sp. *SM1* and in a clinically relevant, pathogenic non-tuberculous mycobacteria (NTM) species, *Mycobacterium heckeshornense*, this genetic locus' believed exclusivity to the MTBC clade is lost. Additionally, species proposed to represent evolutionary intermediates between environmental mycobacteria and professional pathogens, *Mycobacterium riyadhense, Mycobacterium shinjukuense*, and *M. canettii* (Sapriel et al., 2019; Guan et al., 2021), have CRISPR/Cas systems that are distinct from those found in the MTBC, which may suggest that the clonal state of the MTBC is the reason for Type III-A complexes' persistence and that such systems may have already been gained and lost in more mutable mycobacteria. Consequently, relying on non-essential marker loci may be unreliable for tracing evolutionary transitions from within to outside the complex, given the comparative fluidity of NTM genomes. A lack of conservation between spacer sequences across the complexes and species analyzed supports the idea that these systems actively incorporate new spacers in diverse environments and are not inactive relics of an ancestral *Mycobacterium*. Finally, this study expands on the recent findings by Singh et al. (2021) regarding the MTBC, including through the analysis of proximal Cas gene clusters, and also provides a word of caution when inferring ancestry based on the Type III-A system and, indeed, any Cas systems in *Mycobacterium*; the discovery of many seemingly complete and diverse CRISPR/Cas loci across mycobacteria challenges the notion of its scarcity. Furthermore, frequent variation in isolates even within a species supports the idea that mycobacterial horizontal gene transfer plays a large role in the ongoing diversification of these organisms.

## Materials and methods

To collect loci for initial analysis, a database search through CRISPROne and CRISPR-Cas++ was conducted (Zhang and Ye, 2017; Couvin et al., 2018). These databases include the analysis of >32,000 genomes for CRISPROne (2018 release), >36,000 bacterial strains, and >500 archaeal strains for CRISPR-Cas++ (2022 release). These were explored for putative hits in *Mycobacterium*. Most hits in both databases are for *M. tuberculosis* and its variants (e.g., 560 entries out of 786 for CRISPR-Cas++). Questionable matches in each database come with scoring metrics, with most “Evidence Level 1” hits through CRISPR-Cas++'s CRISPRCasFinder being false positives. Potential loci of interest were collected from both databases, though CRISPROne's inclusion of draft genome sequences yielded far more data. Searches were performed with default parameters, except where otherwise noted. Secondary searches of these databases utilized the input of contigs or chromosomes downloaded from NCBI's repository to screen for homologs not included in the CRISPRone and CRISPRCasFinder pre-built databases (Sayers et al., 2022).

To determine the conservation of these sequences across *Mycobacterium* species and to assess any homology hits within the larger context of a locus necessary for actual CRISPR/Cas function, any putative hits in *Mycobacterium* were searched through tblastn and blastn of mycobacterial genomes, including the WGS database for *Mycobacterium* (tax ID: 1763) and subsequently *Mycobacteriales* (Altschul et al., 1990). Expanded searches were also conducted through the Bacterial and Viral Bioinformatics Resource Center website (BV-BRC.org) (Olson et al., 2022). Genomic data uploaded to BV-BRC were annotated through a different pipeline (RASTtk) than NCBI (PGAP), and both were queried for completeness (Brettin et al., 2015; Tatusova et al., 2016).

After the putative detection of two different classes of CRISPR/Cas complexes in some members of the *Mycobacterium kansasii* complex, the complexes were split and searched individually using a similar strategy. TBLASTN queries were conducted using the MK13 Locus I-C Cas8c (a multirole protein in type I-C complexes) and Cas5 (Cas I-C), as well as the MK13 Locus I-G Cas1 (which contains a characteristic Cas4 fusion in I-G complexes) sequences. These queries were performed against the WGS dataset filtered by *Mycobacteriales* (tax ID: 85007) using default parameters. However, the allowed return sequences were increased to 500 to ensure that homologous hits were not prematurely cut off by NCBI filters. Hits were selected from this list for the investigation to determine the likelihood they were real positives and to type any putative CRISPR system(s) identified. Searches were also conducted using PLfams models (genus-specific protein families) through BV-BRC to identify matches in other genomes and assess the genomic context of homologs in other genomes.

CRISPR repeat and spacer sequences were downloaded from CRISPRCasFinder. Owing to the intermittent availability of the CRISPR-Cas++ webserver's CRISPR Repeats and Spacers BLAST tool, the dataset was downloaded from the website, and custom BLAST databases were built on Michigan State University's High-Performance Computing Center supercomputing cluster to query spacers against for conservation using “makeblastdb” in the BLAST+ package (v2.7.1) (Camacho et al., 2009). Using these data, “blastn” searches were performed with default parameters in all cases.

To assess relatedness and clustering of complexes across *Mycobacterium*, a subset of identified Cas1 protein sequences from each annotated or putative complex across were acquired from NCBI and BV-BRC, using CRISPRCasFinder, CRISPROne, and NCBI's ORF Finder to identify loci that had escaped annotation in several cases. The 42 sequences (Supplementary material 1) were aligned using MUSCLE (v3.8.31, all parameters default), which yielded an alignment (Supplementary material 2) of 585 sites (Edgar, 2004). ModelTest-NG was used to calculate the best amino acid substitution model, which recommended LG+G4+F (Supplementary material 3) (Darriba et al., 2020). A maximum likelihood tree was then constructed using RAxML-NG (v.1.0.1) with the following options: –all, substitution model VT+G4+F, seed=882491, –bs-tree autoMRE for automatic determination of bootstrap value convergence. The tree construction process involved 50 random starting trees and 50 parsimony starting trees (Kozlov et al., 2019). The output tree had bootstrap tree support values appended to nodes using raxml-ng –support with the output files from tree generation. The tree was visualized in FigTree v1.4.4, exported as an SVG, and labeled and colored using Inkscape (Andrew Rambaut^1^; Inkscape^2^). The raw tree files in .nwk format are provided as Supplementary material 4.

For the translation of open reading frames and predicted *cas* gene sequences using CRISPRCasFinder, Expasy's Translate tool was used (Gasteiger et al., 2003). For comparison of protein sequence percent identity, the SIM Alignment Tool was used (Gasteiger et al., 2003).

Locus maps were created using BV-BRC's Compare Region Viewer. The exported SVGs were edited using Inkscape software to include only the CRISPR/Cas regions of interest unless other genes were a part of the locus (e.g., transposase insertions) and text labels were applied. The locus maps are only representative and are not drawn to scale. Each locus was visualized using the BV-BRC Compare Region Viewer option set to 20,000 nt.

## Results

### “*cas3*”/TatC

As previously published (He et al., 2012; Singh et al., 2021), very few hits were initially returned for *Mycobacterium* species outside the *M. tuberculosis* complex already known to host a system in either database query. One broadly conserved element that was similar to type-I systems' Cas3 protein was returned but found to be very similar to the non-Cas RNA helicase TatC and subsequently discounted as a false positive.

### Orphan *M. avium* 104 loci

*Mycobacterium avium* strain 104, a well-established CRISPR locus was observed, which has been previously reported in other studies (He et al., 2012; Singh et al., 2021). BLAST searches were performed separately for the direct repeat (DR) sequences and spacer sequences within this locus. These searches utilized custom-built BLAST databases designed for direct_repeat/spacer_seqName.fsa and direct_repeat/spacer_taxon.fsa, specific to CRISPR-CR++ datasets. However, the search results only yielded hits for *M. avium* 104 (Couvin et al., 2018). Additionally, a separate BLASTN search was conducted for the concatenated repeats and spacer sequence of *M. avium* 104: “TGCTCCCCGCGTAAGCGGGGATGAACCGGTCGGTCACTGCGGTGGTGTCCTGTGCATGCTCC.” This search was conducted in the WGS database, filtered by *Mycobacteriales*. The results of this search revealed a match for the complete 13 repeat CRISPR locus in *M. avium* Chester/*M. avium* subsp. *hominissuis* str. ATCC 700898. This particular isolate, identified by ATCC, originated from a human host in 1983 (ATCC).^3^ This timeline corresponds to the isolation of *M. avium* str. 104 in 1983 from a patient in California, USA (Horan et al., 2006), so this match may be spurious. Nevertheless, no *cas* loci were observed in either strain, indicating that they both exhibit an orphaned CRISPR array. When performing a BLASTN search using the *M. avium* 104 CRISPR sequence, along with several thousand flanking bases, against the WGS database for *Mycobacteriales* (taxid 85007), significant hits were obtained with near-complete coverage only in *M. avium* subsp. *hominissuis* strains 101, ATCC 700898, and GM44. Other hits were found in *Mycobacterium fortuitum* and *abscessus*, but they only matched a fragment of the original query (7%−30%). In the case of *M. abscessus* subspecies *massiliense* strain 618 (contig FVWY01000006.1), a partial hit indicated the presence of a different 9-repeat orphan CRISPR locus as idenetified by CRISPRone and CRISPRCasFinder. This locus only showed partial similarity to the locus found in a few *M. avium* strains. Upon comparing the DR sequences and spacer sequences, there was little shared homology, suggesting that these hits are unlikely to be related.

### *Mycobacterium innocens* MK13 loci

**Figure 1:**
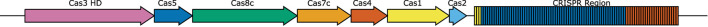
Locus organization of *Mycobacterium innocens* strain MK13 Cas Type I-C system and CRISPR region. The first seemingly complete complex identified in this study, the *Mycobacterium kansasii* complex, was identified in *Mycobacterium innocens* strain MK13 and includes the full expected repertoire of Cas proteins in the usual arrangement for Cas Type I-C systems. The coloring of the CRISPR region indicates direct repeat conservation, with each color representing a unique repeat. Visualization from the BV-BRC Compare Region Viewer and modified in Inkscape.

**Figure 2:**
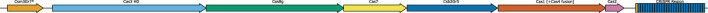
Locus organization of *Mycobacterium innocens* strain MK13 Cas Type “I-U”/I-G system and CRISPR region. A second seemingly complete CRISPR/Cas locus was identified in MK13 but of Cas Type I-G. This locus contains all expected proteins in the usual arrangement for a functional Type I-G system, including the fusion of the Cas4 domain to the N-terminal region of Cas1. The coloring of the CRISPR region indicates direct repeat conservation, with each color representing a unique repeat. Visualization from the BV-BRC Compare Region Viewer and modified in Inkscape.

Two higher-confidence CRISPR loci and upstream arrays of *cas-*like genes were initially identified in the draft genome of *M. kansasii* complex member strain *Mycobacterium innocens* MK13 using CRISPRone. The first complex, comprising 10,974 bp of the 15,118 bp contig UPHQ01000292.1, contained seven *cas*-associated gene homologs in sequence, with a CRISPR locus immediately downstream containing 46 predicted spacers and 47 repeats. As a draft sequence, this was not searchable directly through CRISPRCas++'s CRISPRCasFinder, so the contig UPHQ01000292.1 was downloaded from NCBI and uploaded separately for annotation. CRISPRCasFinder predicts seven generic *cas*-associated genes and, alternatively, four *cas* Type IC–associated genes, along with the same major CRISPR locus of 46 spacers and 47 repeats, showing 0% spacer conservation and 92.61% repeat conservation (Figure 1). While functional labeling is putative without *in vitro* confirmation of activity, the locus will be called locus “I–C” for convenience. The second complex, making up 10,580 bp of the 37,563 bp contig UPHQ01000073.1, was reported using CRISPRone to contain seven *cas*-associated gene homologs in sequence, with another CRISPR locus downstream containing 15 spacers and 14 repeats. CRISPRCasFinder reports this contig contains five *cas*-like genes, annotates 4 as “Type I-U,” and separately annotates 1 gene immediately downstream as Type I-D. The nomenclature for Cas type I-U complexes, “type I, Unknown,” has been updated to Type I-G after initial characterization in recent years (Almendros et al., 2019). As such, this complex is likely to be called the locus or type “I-G” in the future. In type I-G systems, the *cas1* gene, containing a unique fusion of Cas1 and Cas4, is a distinguishing feature (Almendros et al., 2019; Makarova et al., 2020), and this fusion was observed in this study (Figure 2). CRISPRCasFinder also reports 14 spacers and 15 repeats, with 0% spacer conservation and 93.13% repeat conservation. CRISPRCasFinder results are presented in Table 1, and CRISPROne results are presented in Table 2. Tables 3, 4 list the proteins' predicted functional roles that comprise the I-C and I-G loci, respectively.

**Table 1:** CRISPRCasFinder output for *Mycobacterium innocens* MK13 contigs UPHQ01000292.1 and UPHQ01000073.1.

**Coordinates**	**CRISPR/Cas type**	**Number of elements**	**Genes**	**Orientation**
UPHQ01000292.1 3,713–11,095	CAS (generic)	7	*cas2, cas4, cas3, cas3, cas5c, cas7c, cas8c*	Forward
UPHQ01000292.1 5,878–10,803 (overlapping)	CAS Type I-C	4	*cas1, cas5c, cas7c, cas8c*	Forward
UPHQ01000292.1 11,226–14,630	CRISPR Locus	46 spacers	n/a	n/a
UPHQ01000073.1 15,821–22,597	CAS Type I-U	4	*cas3, csb1, csb2, csx17*	Forward
UPHQ01000073.1 22,601–24,241	CAS Type I-D	1	*cas1*	Forward
UPHQ01000073.1 24,696–25,749	CRISPR Locus	14 spacers	n/a	n/a

**Table 2:** CRISPROne output for *Mycobacterium innocens* MK13 contigs UPHQ01000292.1 and UPHQ01000073.1.

**Coordinates**	**CRISPR/Cas type**	**Number of elements**	**Genes**	**Orientation**
UPHQ01000292.1 3,656–11,095	CAS Type I/I-C	7	*cas3, cas5, cas8c, cas7b, cas4, cas1, cas2*	Forward
UPHQ01000292.1 11,226–14,630	CRISPR Locus	46 spacers	n/a	n/a
UPHQ01000073.1 15,169–24,525	CAS Type I/I-U	7	*csm3gr7, cas3, cas8u1, cas7, csb2gr5, cas1, cas2*	Forward
UPHQ01000073.1 24,696–25,749	CRISPR Locus	14 spacers	n/a	n/a

**Table 3:** Components of *Mycobacterium innocens* MK13's Locus I-C Cas complex.

**Coordinates**	**Annotation**	**Activity**	**Association**
UPHQ01000292.1 3,656–5,881	Cas3 HD	Helicase activity (Lundgren et al., 2015) Endonuclease activity (Beloglazova et al., 2011)	CAS complex
UPHQ01000292.1 5,878–6,516	Cas5	crRNA processing (Brendel et al., 2014; Lundgren et al., 2015; Hochstrasser et al., 2016)	Cascade complex
UPHQ01000292.1 6,516–8,264	Cas8c	crRNA processing (Hochstrasser et al., 2016) CAS recruitment (Hochstrasser et al., 2016)	Cascade complex
UPHQ01000292.1 8,266–9,171	Cas7c	crRNA processing (Hochstrasser et al., 2016)	Cascade complex
UPHQ01000292.1 9,164–9,778	Cas4	Exonuclease activity (Zhang et al., 2012)	Spacer acquisition complex
UPHQ01000292.1 9,769–10,803	Cas1	Endonuclease activity (Lundgren et al., 2015) Spacer integration (Lundgren et al., 2015)	Spacer acquisition complex
UPHQ01000292.1 10,805–11,095	Cas2	Structural (Lundgren et al., 2015) Spacer integration (Lundgren et al., 2015)	Spacer acquisition complex

The makeup of this complex classifies it as a complete member of the CAS Type I-C group.

**Table 4:** Components of *Mycobacterium innocens* MK13's Locus I-G CAS complex.

**Coordinates**	**Annotation**	**Functional role**	**Association**
UPHQ01000073.1 15,169–15,681	Csm3Gr7	crRNA processing (Hrle et al., 2013; Lundgren et al., 2015) (Cas7-like)	Type III, interference complex
UPHQ01000073.1 16,001–18,160	Cas3 HD	Helicase activity (Lundgren et al., 2015) Endonuclease activity (Beloglazova et al., 2011)	Type I, CAS complex
UPHQ01000073.1 18,160–20,256	Cas8u/Cas8g^*^	crRNA processing (Lundgren et al., 2015; Shangguan et al., 2022) CAS recruitment (Shangguan et al., 2022)	Type I, cascade complex Type I, interaction between Cascade and CAS Complexes
UPHQ01000073.1 20,253–21,212	Cas7	crRNA processing (Shangguan et al., 2022)	Type I, cascade complex
UPHQ01000073.1 21,215–22,597	Csb2gr5	crRNA processing by Cas5/Cas6 fusion activity (Shangguan et al., 2022)	Type I, cascade complex
UPHQ01000073.1 22,601–24,241	Cas1	Endonuclease activity (Lundgren et al., 2015) Spacer integration (Lundgren et al., 2015) Cas4 nuclease activity (Almendros et al., 2019)	Type I, spacer acquisition complex
UPHQ01000073.1 24,238–24,525	Cas2	Structural (Lundgren et al., 2015) Spacer integration (Lundgren et al., 2015)	Type I, spacer acquisition complex

In 2019, the CAS Type I-U (for “unknown”) complex was renamed to Type I-G after it was found that Cas1 in this class is a fusion of Cas4 and Cas1 domains, but the exact roles of proteins in this complex remain poorly understood. The makeup of this complex classifies it as a complete member of the CAS Type I-G group, but Csm3 does not play a known role in this system, and its presence here is atypical. CRISPRCasFinder does not predict this ORF, and subsequent searches suggest this may be a false positive.

### *Mycobacterium innocens* MK13 CRISPR sequences for discovery

To explore whether related species have also acquired or maintained these CRISPR loci, a BLAST search of the more stringent NR database with DR sequences from *M. innocens* MK13 was performed but yielded no matches. A subsequent search of the WGS database filtered by organisms in the taxon *Mycobacterium* (tax ID: 1763) yielded some significant hits (Table 5). Before proceeding, the *Mycobacterium persicum* MK4 contig UPHM01000048.1 exhibited a 100% match to DR sequences. This contig was subsequently uploaded to CRISPR-Cas++ and searched using CRISPRCasFinder. The search results revealed the presence of a homologous set of *cas*-like genes and a CRISPR locus containing 50 spacers, which included the DR sequence identified through the initial BLAST analysis. Despite the high conservation of DR sequences between these two members of the *M. kansasii* complex, the spacer sequences were found to be unique for both. A BLAST search of the WGS database filtered by *Mycobacterium* (tax ID: 1763) with the locus I-C DR consensus yielded 22 hits. Similarly, when the same strategy was applied using the locus I-G, 24 hits (Table 5). Some hits matched multiple contigs from the same sequencing run (e.g., Table 5, *M. persicum* AFPC-000227 with four separate contigs). Hits for I-C were observed in *M. persicum* (10/15), *M. innocens* (4/15), and one from *Mycobacterium attenuatum* (1/15). Expanding the search to *Actinobacteria* (tax ID: 201174) only added one additional hit in *Mycobacterium* sp. SM1, an environmental isolate from a mud volcano in Italy (Korea Institute of Geoscience Mineral Resources, 2021). Contigs for this organism were downloaded and searched using CRISPRCasFinder, which returned the aforementioned I-G locus and an unexpected second locus on a separate contig. Other hits for I-G included *M. canettii* (8/20), *M. riyadhense* (4/20), *M. innocens* (4/20), *Mycobacterium ostraviense* (2/20), and *Mycobacterium gastri* (2/20). Expansion of this DR to *Actinobacteria* yielded no additional hits. The secondary locus identified on *Mycobacterium* SM1 contigs was annotated by CRISPROne and CRISPRCasFinder as a type III-A CRISPR/Cas system.

**Table 5:** BLASTN results for *Mycobacterium innocens* MK13 direct repeat (DR) consensus sequences from a locus on UPHQ01000292.1 (“locus I-C”) and a locus on UPHQ01000073.1 (“locus I-G”) identified by CRISPRCasFinder searched against the WGS database filtered to include species in *Mycobacterium* (tax ID: 1763).

**DR origin**	**Strain**	**Query coverage (identity)**	**Contig accession number**
MK13 locus I-C	*Mycobacterium innocens* MK13 **(source)**	100% (100%)	UPHQ01000292.1
MK13 locus I-C	*Mycobacterium persicum* MK4	100% (100%)	UPHM01000048.1
MK13 locus I-C	*Mycobacterium persicum* MK42	100% (100%)	UPHL01000057.1
MK13 locus I-C	*Mycobacterium persicum* MK15	100% (100%)	UPHK01000053.1
MK13 locus I-C	*Mycobacterium innocens* 49/11	100% (100%)	NKRC01000001.1
MK13 locus I-C	*Mycobacterium persicum* 12MK	100% (100%)	MWQA01000001.1
MK13 locus I-C	*Mycobacterium persicum* 7MK	100% (100%)	MWKZ01000001.1 MWKZ01000001.1
MK13 locus I-C	*Mycobacterium persicum* 3MK	100% (100%)	MWKX01000001.1
MK13 locus I-C	*Mycobacterium persicum* 8MK	100% (100%)	MWKV01000001.1
MK13 locus I-C	*Mycobacterium persicum* AFPC-000227	100% (100%)	MVIF01000358.1 MVIF01000183.1 MVIF01000100.1 MVIF01000083.1
MK13 locus I-C	*Mycobacterium persicum* 1010001469	100% (100%)	LWCM01000086.1
MK13 locus I-C	*Mycobacterium innocens* 1010001493	100% (100%)	LWCK01000074.1
MK13 locus I-C	*Mycobacterium innocens* 1010001454	100% (100%)	LWCH01000340.1
MK13 locus I-C	*Mycobacterium persicum* CSURQ1465	100% (100%)	CADEAW010000279.1 CADEAW010000263.1 CADEAW010000143.1CADEAW010000058.1
MK13 locus I-C	*Mycobacterium attenuatum* MK41	100% (97.30%)	UPHT01000066.1
MK13 locus I-G	*Mycobacterium innocens* MK13 (source)	100% (100%)	UPHQ01000073.1
MK13 locus I-G	*Mycobacterium ostraviense* 241/15	100% (100%)	NKRE01000001.1
MK13 locus I-G	*Mycobacterium innocens* 49/11	100% (100%)	NKRC01000001.1
MK13 locus I-G	*Mycobacterium innocens* 1010001493	100% (100%)	LWCK01000025.1
MK13 locus I-G	*Mycobacterium ostraviense* 1010001458	100% (100%)	LWCI01000115.1
MK13 locus I-G	*Mycobacterium innocens* 1010001454	100% (100%)	LWCH01000288.1
MK13 locus I-G	*Mycobacterium gastri* DSM 43505	100% (100%)	LQOX01000131.1
MK13 locus I-G	*Mycobacterium riyadhense* MR-246	100% (100%)	CAJMWP010000001.1
MK13 locus I-G	*Mycobacterium riyadhense* MR-244	100% (100%)	CAJMWO010000001.1
MK13 locus I-G	*Mycobacterium riyadhense* MR-206	100% (100%)	CAJMWK010000001.1
MK13 locus I-G	*Mycobacterium riyadhense* MR-1023	100% (100%)	CAJMWI010000001.1
MK13 locus I-G	*Mycobacterium gastri “*Wayne”	100% (100%) 100% (100%) 100% (100%)	AZYN01000299.1 AZYN01000281.1 AZYN01000120.1
MK13 locus I-G	*Mycobacterium canettii* STB-K	94% (97.06%) 94% (97.06%)	JAHVHL010000003.1 JAHVHL010000002.1
MK13 locus I-G	*Mycobacterium canettii* NLA000701671	94% (97.06%)	JACTAP010000068.1
MK13 locus I-G	*Mycobacterium canettii* CPIT 140070013 (2013)	94% (97.06%)	CAON01000366.1
MK13 locus I-G	*Mycobacterium canettii* CPIT 140070002	94% (97.06%)	CAOL01000412.1
MK13 locus I-G	*Mycobacterium canettii* CPIT 140070013 (2022)	94% (97.06%)	CAMJXS010000071.1
MK13 locus I-G	*Mycobacterium canettii* Percy1101	94% (97.06%)	CAKKKT010000093.1
MK13 locus I-G	*Mycobacterium canettii* Percy258	94% (97.06%)	CAKKKM010000107.1
MK13 locus I-G	*Mycobacterium canettii* Percy525	94% (97.06%)	CAKKKA010000091.1
MK13 locus I-G	*Mycobacterium canettii* CIPT 140070002	94% (97.06%)	CAJJDU010000059.1

A BLASTN megablast search was conducted on *M. canettii* (tax ID: 78331) using the seven DR sequences from locus I–C. The search was performed with low-complexity filtering turned off. Modest-confidence hits were obtained in *M. canettii* strain STB-K, where multiple contigs matched for five out of the seven DR sequences. One specific contig (JAHVHL010000002.1) has four closely located matches and was subsequently analyzed using CRISPRCasFinder, along with the other two major contigs. This analysis identified a homologous I-C CRISPR/Cas region with 53 spacers. However, in this genome, a second CRISPR locus with 28 spacers was identified. The DR sequences within this locus exhibited 99.4% conservation. It is worth nothing that this second CRISPR locus was located ~240,000 bp away from the nearest predicted *cas* genes and was positioned directly at the end of the contig (coordinates: 970,599–972,654, contig length: 972,672). The repeat consensus was unique among those located proximal to the *cas* locus, and an identical match to this alternate DR sequence was found at the start of another contig in the assembly (NZ_JAHVHL010000003.1, coordinates: 27–851 bp). Searching the contigs again using the CRISPRCasFinder with the “unordered” option enabled allowed for the identification of *cas* gene hits that do not include a complete and ordered locus. This search revealed genes starting at position 1,023 bp, including an untyped cas2, a cas 1 gene belonging to a specific type ID, and a sequence of four genes forming a “type IU.” A literature search indicated that a resequencing study conducted by Blouin et al. in 2014 had previously identified the presence of two systems in STB-K. However, in their classification, one system was classified as Type I-C, while the other was labeled as a Type I-C variant. Moreover, it is worth noting that at the time of their publication, type I-G systems had not been characterized (Blouin et al., 2014). With evidence of various existing CRISPR/Cas systems across the genus, we expanded the search to incorporate Cas protein sequences for system discovery rather than relying on the identification of CRISPR repeats, which can be difficult to sequence and assemble and may not be identified reliably.

### *Mycobacterium innocens* MK13 Cas sequences for discovery

Using BV-BRC's PLfams browser to search for the motif of the *cas2*-like gene in *M. innocens* MK13 and searching genus-specific families for hits in the *Mycobacterium* genus, two strains of reference or representative quality were found to possess loci showing structural homology to the putative MK13 CRISPR/Cas locus: these strains are *M. gastri* DSM 43505 and *M. ostraviense* FDA-ARGOS_1613. These two species have recently been reclassified as members of the *M. kansasii* complex of organisms (Jagielski et al., 2020). Upon conducting a search of the *M. ostraviense* FDA-ARGOS_1613 assembly using CRISPRCasFinder returns, we found the presence of a 127-spacer CRISPR array with 97%+ DR conservation. However, CRISPRCasFinder did not identify the adjacent conserved locus as a Cas cluster in the same manner as it did for *M. kansasii* MK13. In contrast, the CRISPROne tool was able to identify both the CRISPR array and the adjacent 5-gene Cas clusters.

**Figure 3:**
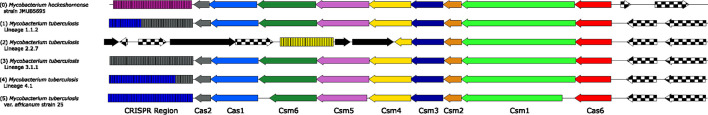
Comparison of the Cas Type III-A system in *Mycobacterium heckeshornense* strain JMUB5695 vs. MTBC members. The discovery of a Cas Type III-A system outside the *M. tuberculosis* complex prompted an investigation into the similarity between it and examples inside the complex. Genes with checkerboard coloring are unrelated to the complex, and genes colored solid black are transposase sequences. A comparison of Cas10 (Csm1, bright green) sequences between *M. heckeshornense* and the MTBC isolates above showed 72%−73% identity using Expasy's SIM tool. When comparing *M. heckeshornense* to the insertion element-truncated Cas10 in *Mycobacterium* sp. *SM1*, overlapping regions shared 78% identity. Finally, comparing *SM1* Cas10 to *MTB* Cas10 yielded 70%−71%.

A TBLASTN of the MK13 Type I-G Cas1 protein returned hits in two broad groups: a high similarity group (75%+ AA identity) and a partial similarity group (~26%−40% AA identity). High-similarity homologs were identified in *M. kansasii* complex strains *gastri, ostraviense*, and *innocens*, as well as in *M. canettii* and *M. riyadhense*. Partial similarity homologs were identified again in the aforementioned *M. kansasii* complex strains, a wide group of genera outside *Mycobacterium* (*Corynebacterium, Nocardia, and Rhodococcus*) and in *M. tuberculosis* variant *tuberculosis*. High-similarity homologs showed 99%+ query coverage against Type I-G Cas1, and partial similarity groups showed roughly 50%−70% query coverage. Several hits did not belong to these categories, such as 99% coverage/47%−60% identity for *Mycolicibacterium hassiacum, Prescottella subtropica, Gordonia paraffinivorans*, and others. Of this set, *M. heckeshornense* featured two sets of hits, one with 68% coverage and one with 31% coverage. In the case of *M. heckeshornense* strain DSM 44428, the contigs JACKTA010000047.1 and JACKTA010000078.1 were downloaded from NCBI in FASTA format. These contigs were then searched using CrisprCas++, and although contig 47 is short (4,529bp), it was predicted to contain a CRISPR locus with 36 spacers, as well as the presence of *cas2* and a partial *cas1* gene. Contig 78 (86,845 bp) was found to contain a predicted complex close to the end of the locus. However, this complex contains some predicted *cas* genes of Type I-U, according to CRISPRCasFinder. When using CRISPROne, the same system was identified for contig 47, but the complex on contig 78 was not considered credible enough to make a definite call. The second *M. heckeshornense* WGS dataset (strain RLE) had the same two hits, and interestingly, the two contigs bearing these hits were nearly the same lengths (85,024 and 4,395 bp, respectively). Unfortunately, SRA data for the two *M. heckeshornense* assemblies are publicly unavailable, and reconstruction of these possible loci is not possible now. However, the complete genome of *M. heckeshornense* JCM 15655 was searched next, and a locus disrupted by a transposase and with the structure of [*csb1-csb2-cas3-*hypothetical protein- < IS1380>-*cas1-cas2*] was extracted for sequence comparison. A second locus was not evident in this genome. An additional genome, *M. heckeshornense* JMUB5695, was also identified to contain a high-confidence Cas Type III-A locus, significantly similar to the complex inside the MTBC and some *M. canettii* strains (Figure 3). The same genome also contained a second locus similar to the order observed in *M. heckeshornense* JCM 15655, *Mycobacterium marinum* VIMS9, and one in *M. shinjukuense* JCM 14233. However, this locus includes an unannotated hypothetical protein within the same frame as the other genes that form the *cas* locus.

A search for *M. hassiacum* returned two hits in CRISPRCasFinder. This genome was reported to contain a CRISPR array by Singh et al. (2021), but both *cas* loci presented with an atypical structure of *cas3-csb2-csb1-hypothetical protein-cas1-cas 2*. This hypothetical protein contains no detected conserved domains and fits neatly between *csb1* and *cas1* in both loci. It is unclear whether this system is active, but both are included in the concatenated Cas FASTA file (Supplementary material 1). The first *cas* locus contains 44 CRISPR spacers downstream, and the other has a severely disrupted CRISPR locus punctuated with four repeats of IS6120 transposases between short CRISPR repeats before an intact 19-spacer array. This same atypical *cas* locus structure with a protein of unknown function in the operon was also found in *Mycobacterium longobardus* DSM 45394, with a 54-spacer CRISPR locus in place downstream, which may represent a novel cas-like gene.

**Figure 4:**
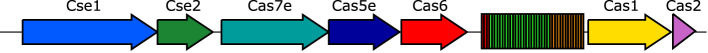
Structure of the *Mycobacterium austroafricanum* Cas locus. Species *austroafricanum* presented with an arrangement of *cse* genes upstream of the CRISPR locus and the Cas1-Cas2 cluster downstream.

*Mycobacterium austroafricanum* DSM 44191 presented another interesting arrangement. This genome was also reported to contain a CRISPR array (Singh et al., 2021) and appeared to have both an atypical Cas locus and a homolog in *Mycobacterium* sp. *D3*. A full repertoire of five *cse* was found upstream of the CRISPR array, and *cas1* and *cas2* were found immediately downstream of it (Figure 4).

Some *M. xenopi* strains may have two systems, but one with a Cas1/4 fusion homolog was split across multiple contigs. The complete system (Cas Type I-E) was upstream of a reported CRISPR repeat region (Singh et al., 2021), but the putative type I-G system was not recoverable and could not be reconstructed from the available sequence.

*Mycobacterium vanbaalenii* strain JOB5 did not appear to have a chromosomal Cas system. However, an unusual locus was identified by both CRISPROne and CRISPRCasFinder (the latter with the search option “unordered”). This locus featured homologs of *csf1, csf4, csf2, and csf3*, which were found ~10 kb upstream of a small, low-evidence 3 spacer CRISPR locus. Such a finding may very well be noise, but it does share similarities with wildly divergent, plasmid-borne Type 4 Cas systems (Pinilla-Redondo et al., 2020). A TBLASTN search of the NR database for the four concatenated genes returned 95 hits, nearly all of which were on plasmids. Representative sequences were taken from a *Mycobacterium fluoranthenivorans* 2A plasmid (CP059893.1), *Mycobacterium* sp. *YC-RL4* plasmid pMYC1 (CP015597.1), and the chromosomal sequence of *Mycobacterium rhodesiae* NBB3. Although confidence in these sequences making up a complete Type IV system was low, these systems are known to be highly divergent even within subtypes. The challenge lies in their identification caused by the lack of reliable signature genes. Therefore, these sequences are still being included in the analysis with appropriate caution. It is essential to exercise prudence when interpreting the results without *in vitro* confirmation of their functional implications.

*Mycobacterium riyadhense* MR-246 appeared both in the Locus IG Cas1 TBLASTN search as well as the search using the MK13 DR sequence, with both hits independently finding the same region in the chromosome (CAJMWP010000001.1). CRISPRCasFinder and CRISPRone independently identified a 111-spacer CRISPR array with 97% DR conservation and 0% spacer conservation, although only CRISPRone identified upstream *cas* genes. The subtype on this chromosome was reported as I-U (I-G), but the locus was missing a *cas2* gene call. While re-running CRISPRCasFinder and enabling the “Unordered” option to allow for hits outside a longer locus, it also returned the same hits as those by CRISPROne, but it was also missing a *cas2* gene call, which presumably led this software to initially discard the other five genes upstream of the CRISPR array. NCBI's ORF Finder output from the full locus (4,227,944–4,244,859, 16,916 bp) identified a 95aa open-reading frame directly downstream of *cas1* and upstream of the CRISPR array, and a BLASTP search of this protein against the UniProtKB database identified 17 moderate confidence alignments (*E*-value range = 6^−11^ to 0.003) against Cas2 proteins. No other hits were reported. NCBI's Conserved Domain Database (CDD) search of query protein sequences against a database of known domains returned a hit on a Cas2 domain in this sequence (from position 5–70, *E*-value = 5.54^−17^). Given that the divergence of the *cas2*-like ORF in *M. riyadhense* led to CRISPRCasFinder missing the entire locus and CRISPROne identifying 5/6 genes plus the CRISPR array but not the *cas2* homolog, the entire nucleotide sequence of the locus from ORF Finder was also used for an unfiltered BLASTN search of the nt database and a broad scan of the WGS database filtered by hits in *Actinomycetota*. The NR database returned a hit with 16,914 nt of coverage and only a single non-identical nucleotide from *M. riyadhense* strain NTM, as well as high confidence hits (*E*-value = 0.0) in *M. ostraviense* str. FDAARGOS 1613 and *M. canettii* strains CIPT 140070010, 140070008, and 140070017.

*Mycobacterium lacus* JCM 15657 presented with a striking 146-repeat CRISPR locus, and subsequent investigation showed unannotated upstream *cas2* and *cas1* genes, but the rest of the locus appeared unusual, with GenBank annotation noting frameshifts present. A BLASTP search of the sequence upstream of Cas1 hit numerous IS3 family transposases scattered throughout *Mycobacterium lacus, kansasii*, and *persicum* strains, as well as in other genera. It appears that this may have been a functional system broken by the insertion of this transposase in the past, and a scan of similar disruptions finds an IS3 family transposase where Cas1 and Cas2 would be found in the CRISPR/Cas locus of *M. tuberculosis* strain 36918. This disruption appears widespread across Beijing-like strains (as can be seen in the lineage 2 isolate in Figure 3), with a BLASTN search of the locus returning dozens of nearly identical sequences with 100% query coverage containing the disrupted region from other MTB Beijing-like isolates.

**Figure 5:**
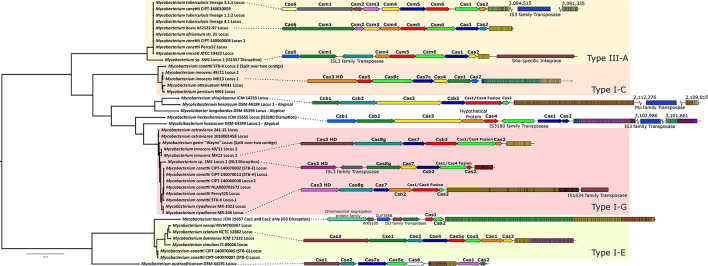
Phylogeny and organization of putative Cas systems across *Mycobacteriaceae*. **Left:** phylogenetic tree of mycobacterial Cas1. A maximum likelihood tree was generated out of 42 Cas1 protein sequences (detected either by existing annotation, CRISPROne/CRISPRCasFinder annotation, and/or NCBI Conserved Domain Search annotation). Sequences were aligned by MUSCLE, and a phylogenetic tree was generated by RAxML-NG with bootstrapping. Branches are drawn to scale, with the scale bar representing average amino acid substitutions per site. Bootstrap values are not shown but are available in Supplementary material 4. The tree is visualized and rooted at the midpoint by FigTree. Raw tree data are provided in Supplementary material 4 for transparency. **Right:** representative locus maps of identified Cas systems. Organization of the types of Cas complexes identified in this work. Locus maps created through the BV-BRC Compare Regions Viewer. Dashed lines from the tree indicate the genetic origin of each locus shown, and genes are labeled as described above. Complexes are grouped into their existing types when possible (e.g., Type I-G). CRISPR repeat sequences are indicated by colored rectangles, with each box indicating a spacer and repeat and differently colored boxes representing a specific CRISPR repeat sequence within the locus. In most cases, flanking genes are removed from the visualization for clarity, but transposases/integrases/insertion sequence elements are labeled and included. Both tree and locus visualizations are not exhaustive or comprehensive in coverage, and other loci and arrangements across *Mycobacteria* exist. Figure created in Inkscape.

The phylogenetic tree of 42 representative mycobacterial Cas1 protein sequences yielded broad clustering along expected lines based on existing Cas classifications (e.g., Type I-C, Type I-E, etc.; Figure 5). Some disrupted or divergent putative Cas systems fell outside these clusters even when analyzed by their intact Cas1 sequences.

## Discussion

Despite longstanding knowledge that CRISPR/Cas systems are present in *M. tuberculosis*, a species with very limited horizontal gene transfer compared to other bacteria (Panda et al., 2018), an investigation into the prevalence of these systems across other mycobacteria has been limited. In recent years, some studies have been conducted, such as the study by Singh et al. (2021), which explored the extent of CRISPR/Cas systems outside the MTBC, but their reasonably cautious focus on completed genomes and CRISPR arrays rather than complete CRISPR/Cas gene sets limited the scope of potential discovery. Using available software such as CRISPROne and CRISPRCasFinder in combination with a more fine-toothed approach of manually investigating many draft genomes and checking for ORFs missed by annotation packages has revealed a substantial and previously unreported spread of multiple Cas types across *Mycobacterium*, ranging from free-living organisms to pathogens.

These *in silico* data show that high-confidence homologs to CRISPR/Cas systems exist outside the MTBC, but they do not prove this without functional validation. Despite CRISPROne and CrisprCas++ algorithms being designed to minimize false positives, the widespread annotation of a TatC homolog as an orphan Cas3 protein in *Mycobacterium* is a reminder that all output requires cautious interpretation.

### The *M. avium* complex does not contain Cas loci

The CRISPR locus identified in *M. avium* strain 104 was not found in any other *M. avium* complex species in the NCBI NR database. As observed in large sequence polymorphisms that contain unique genetic elements not observed in other mycobacteria, such as those involved in metal acquisition and regulation in *M. avium* subspecies *paratuberculosis* (Semret et al., 2004; Bannantine et al., 2012), members of the MAC do have the potential to take up foreign genetic elements. Similarly, recent research has begun to elucidate the frequency and importance of HGT in *Mycobacterium* despite its limits in some species (Panda et al., 2018). Identifying a homologous sequence containing this orphan CRISPR in other closely related (or possibly identical, in the case of *M. avium* ATCC 700898) *M. avium* isolates at least minimizes the likelihood that it is a sequencing error. However, the closest match outside these immediate relatives of *M. avium* isolates appears to be a different orphan CRISPR in *M. abscessus* subspecies *massiliense*. Regardless, the origins of these orphan CRISPR loci remain unknown. No further evidence of any *cas* or *cas*-like genes was identified in any *M. avium* complex species, which appears to be devoid of these loci except for this orphaned CRISPR element in a single strain.

### Other phylogenetically diverse mycobacterial taxa contain numerous diverse CRISPR/Cas systems

This study demonstrates the prevalence and diversity of CRISPR/Cas systems in the clinically important *M. kansasii* complex of organisms. *M. innocens* MK13's locus I-C showed homologs within the *M. kansasii* complex, with most concentrated in *M. persicum*, a recently characterized opportunistic pathogen in the complex originally isolated from sputum in multiple cases of human disease in Iran (Shahraki et al., 2017). Other hits were in species that are not considered pathogenic: *M. innocens* and *M. attenuatum* (Tagini et al., 2021).

In contrast to this grouping within the MKAN complex, MK13's locus I-G returned a surprising number of hits in pathogenic mycobacteria outside the complex: 8 in *M. canettii*, the smooth tubercule species at the edge of the *M. tuberculosis* complex; and 4 in *M. riyadhense*, a human NTM pathogen first identified in Saudi Arabia (van Ingen et al., 2009). The other hits were in *M. innocens, M. gastri, M. ostraviense*, and *Mycobacterium* sp. *SM1* is largely a non-pathogenic or opportunistic pathogen organism (Tagini et al., 2019, 2021) and an environmental isolate in the case of *SM1* (Awala et al., 2022). *Mycobacterium innocens*, it seems, possesses two potential CRISPR/Cas systems that bifurcate either within the MKAN complex or outside of it.

Existing literature can tell us much about the composition of the complexes identified in this study. Cas5 is involved in pre-crRNA processing into crRNA units (Brendel et al., 2014; Lundgren et al., 2015; Hochstrasser et al., 2016). In Cas type I systems, Cas6 typically functions with Cas5 and Cas7 in crRNA processing, but Type I-C systems are unique in that Cas6 is absent, Cas5 functions on its own, and Cas8c is a signature of these types of loci (Garside et al., 2012; Brendel et al., 2014; Lundgren et al., 2015; Hochstrasser et al., 2016), streamlining the Cascade complex and allowing full functionality with only Cas5, Cas7c, and Cas8c, all of which are intact in MK13 locus I-C. This suggests that, at a minimum, *M. innocens* MK13 possesses an intact ability to process pre-crRNA into its target-binding form for a Cas complex. Similarly, type I-G systems have a fusion of Cas1 and Cas4 (Shangguan et al., 2022), which is also observed in MK13's locus I-G *cas1* gene, showing an N-terminal Cas4 domain and a C-terminal Cas1 domain per NCBI's CDD search. This provides a complete complex of Cas3, Cas8, Cas7, Csb2, Cas1 (with the Cas4 fusion), and Cas2.

The *M. riyadhense* MR-246 CRISPR/Cas locus identified by both CRISPR repeat sequence and type I-G Cas1 BLAST searches is an interesting example where the stringency of software to eliminate false positives is likely obscuring the identification of new features. The comparatively low percent identity values of this ORF (28%−49%) combined with the short sequence length of Cas2 proteins (Samai et al., 2010) likely contribute to both the relatively modest *E*-values and the exclusion of this divergent Cas2 protein from identification by both software tools. The nearly identical loci (one mismatch out of 16,914bp) between *M. riyadhense* MR-246 and *M. riyadhense* NTM raise questions, and BioSample records (SAMEA7003857, SAMN12495011) indicate that the samples were collected in 2016 and 2018, respectively, from human disease cases in Saudi Arabia. An additional 2,000 nt on each side of the *M. riyadhense* NTM locus aligned to MR-246 was extracted, and a BLAST search was run against other *riyadhense* isolates to assess how much variation occurs between other clinical isolates and to assess if additional CRISPR spacers had been acquired in the newer 2018 NTM isolates using CRISPROne. The same number of spacers were identified, and differing numbers were observed in other *riyadhense* isolates. This high similarity may indicate that *M. riyadhense* isolates MR-246 and NTM originated from a common point of exposure, but this is beyond the scope of this investigation. *Mycobacterium riyadhense* has been proposed as an intermediate between environmental and professionally pathogenic mycobacteria in the MTBC (Guan et al., 2021). It is tempting to speculate about following the Type I-G breadcrumbs down an evolutionary path from environmental organisms such as *M. innocens*, through *riyadhense*, toward the *prototuberculosis*-analog of *canettii*, and finally into *M. tuberculosis*, and it has been reported that the Type I-G-bearing *M. canettii* STB-K is one of the most distant from other *canettii* members (Supply et al., 2013). However, a Type I-C complex was observed in STB-K and was not discovered in the MTBC or other proposed evolutionary stepping-stone species like *M. shinjukuense* or *M. riyadhense* (Sapriel et al., 2019; Guan et al., 2021), so tracing any such path is complicated and risky.

### The type III-A Cas system is not exclusive to the *M. tuberculosis* complex

In searching for homologs for type I-G complexes, the curious case of *Mycobacterium* sp. *SM1* arose, which was unexpectedly found to contain a disrupted form of the Cas Type III-A system previously reported to exist exclusively in the MTBC and some isolates of *M. canettii* (Singh et al., 2021). An analysis of the operon arrangement suggests that SM1's locus was missing *csm3* and *csm2*, and its *csm1* gene was truncated, with the area disrupted by an IS3 family-like transposase. TBLASTN searching for this transposable element returned a long range of matches to *M. lacus, M. riyadhense*, and numerous *M. tuberculosis* complex strains. In MTB H37Rv, its homolog is annotated as a possible IS1557 transposase (mycobrowser.epfl.ch/genes/Rv1313c). Little is currently known about *M*. sp. *SM1*. However, it is annotated as having been discovered in an Italian mud volcano (Korea Institute of Geoscience Mineral Resources, 2021), and an abstract from the International Meeting of the Microbiological Society of Korea in October of 2022 by Awala et al. (2022) describes the species as an “extremely acidophilic” bacterium, growing in an optimal pH of 2–4. How this organism or its ancestors could have either received or donated this Cas system to other mycobacteria is another fascinating open question, but one beyond the scope of this study. However, in exploring the homology of this complex to other Type III-A systems in *Mycobacterium*, another homologous but intact Cas Type III-A locus was found in *M. heckeshornense* JMUB5695, with an arrangement nearly identical to that observed in the MTBC except for spacer variation in its two downstream CRISPR arrays. This organism is known to cause severe pulmonary disease in immunocompetent adults worldwide (Van Hest et al., 2004; Coitinho et al., 2016; Iitoh et al., 2020).

### CRISPROne reports the homology of conserved, plasmid-borne genes in some mycobacteria and unusual type IV Cas systems

Finally, and with a word of caution, some mycobacteria may possess Type IV Cas systems. The abundance of four clustered genes highlighted by CRISPROne as potential homologs to Type IV proteins across mycobacterial plasmid sequences from many different species suggests that those mycobacterial plasmids may themselves be involved in targeting other plasmids (Pinilla-Redondo et al., 2020). However, these systems are notoriously difficult to predict, and while this merits further follow-up, it is not sufficient to assign these loci as Type IV Cas systems purely by computational analysis.

## Conclusions

In summary, this study has revealed numerous mycobacterial CRISPR/Cas systems. Rather than being an oddity or an indicator of the genetic rigidity of the *M. tuberculosis* complex, these systems are frequent across environmental species, opportunistic pathogens, and professional pathogens. However, the absence of such systems in genetically close organisms is strange; in the *M. kansasii* complex, *M. kansasii* and *M. pseudokansasii* were not observed to contain *cas* genes, even though the former has even been reported to be a DCT recipient from both *M. persicum* and *M. attenuatum*, CRISPR/Cas bearing species within the complex (Tagini et al., 2021), or even the variation in the presence or type of systems within the same species, as was previously observed in *M. canettii* species but has now been expanded to across *Mycobacterium*. The presence or absence of one or more systems in one organism seems to have little bearing on whether all members of that species or its closest relatives will have the same loci. However, one pattern did emerge: some broad groups of mycobacteria do not appear to have CRISPR/Cas systems.

Barring an orphan CRISPR locus in *M. avium* 104, also called *M. avium* ssp. *hominissuis* (MAH), no evidence of any Cas proteins, other CRISPR arrays, or even variation in the MAH CRISPR locus was found across any member of the *M. avium* complex. Through BV-BRC, a search of 493 *M. avium* genomes for Cas domains returned only a single partial hit for *cas2* on a 521bp contig from a study characterizing hypervariable genomic islands from German MAH strains (Sanchini et al., 2016). While it is now understood that many mycobacteria undergo horizontal gene transfer by DCT, this form of exchange remains poorly elucidated. Since systems exist sporadically within even tight phyletic groups, it may be that CRISPR/Cas systems are not ancestral to mycobacteria, at least in recent history.

Instead, horizontal gene transfer could be a primary mechanism through which CRISPR/Cas systems are acquired in mycobacteria. It is known that plasmids carry CRISPR/Cas systems (Pinilla-Redondo et al., 2022) and both the systems on plasmids and genomic systems often target mobile genetic elements such as plasmids (Pinilla-Redondo et al., 2020; Wheatley and MacLean, 2021). The rarity of mycobacterial plasmids has been noted (Gray and Derbyshire, 2018), and plasmids that do transfer between species are also compartmentalized, as reported by Ummels et al., who reported the discovery of a conjugative plasmid capable of exchange between slow-growing mycobacteria. However, this unique plasmid was unable to be transmitted to any fast-growing mycobacteria (Ummels et al., 2014). While plasmids are still believed to be important and do play key roles in mycobacteria (Dumas et al., 2016), the mechanism of DCT, where chromosomal DNA is transferred between mycobacterial cells, appears to be the predominant means of horizontal gene transfer (Gray and Derbyshire, 2018). This unusual meiotic-like means of genetic exchange, the frequency of transposable elements across mycobacteria, and a low frequency of other gene transfer mechanisms could explain the inconsistent but still widespread prevalence of various CRISPR/Cas systems across mycobacteria. Additionally, both the disruption of the Cas system in hypervirulent *M. tuberculosis* lineage 2 isolates and the numerous instances of mobile genetic elements having already disrupted newly discovered loci herein indicate that CRISPR/Cas systems do not appear essential to most mycobacterial lifestyles.

While their historical use in tracing the clonality and evolution of *M. tuberculosis* preceded even an understanding of their function, it would seem that CRISPR/Cas systems are not suited to direct evolutionary extrapolations. Instead, their presence and absence appear, like mycobacteria themselves, enigmatic, implying both a frequent flow of genetic material in and out of most mycobacteria and that the NTM pangenome may substantially expand as more isolates are sequenced. Future studies should confirm the functionality of these newly reported systems, including whether several phylogenetically diverse systems, such as hypothetical proteins, may represent novel Cas types or functions.

## Data availability statement

The datasets presented in this study can be found in online repositories. The names of the repository/repositories and accession number(s) can be found in the article/Supplementary material.

## Author contributions

SS obtained funding, helped design the study, interpreted the data, and edited the manuscript. EB performed the work, analyzed genomes, organized data for visualization, and wrote the manuscript drafts. All authors contributed to the article and approved the submitted version.

## Footnotes

^1^Andrew Rambaut. *FigTree*. http://tree.bio.ed.ac.uk/software/figtree/ (accessed April 3, 2023).

^2^Inkscape. *Inkscape Project Open-source Graphics Editor*. Available online at: https://inkscape.org/ (accessed April 3, 2023).

^3^ATCC *Mycobacterium avium* strain Chester. https://www.atcc.org/products/700898 (accessed April 3, 2023).

## References

[B1] Almendros C. Nobrega F. L. McKenzie R. E. Brouns S. J. J. (2019). Cas4-Cas1 fusions drive efficient PAM selection and control CRISPR adaptation. Nucleic Acids Res. 47, 5223–5230. 10.1093/nar/gkz217 30937444 PMC6547450

[B2] Altschul S. F. Gish W. Miller W. Myers E. W. Lipman D. J. (1990). Basic local alignment search tool. J. Mol. Biol. 215, 403–410. 10.1016/S0022-2836(05)80360-2 2231712

[B3] Awala S. I. Gwak J. H. Seo C. Bellosillo L. A. Kim Y. -M. Si O. -J.. (2022). “Short-chain alkanes consumption in Mycobacterium sp. SM1 isolated from an acidic geothermal environment,” in MSK 2022: International Meeting of the Microbiological Society of Korea (Seogwipo).

[B4] Bannantine J. P. Wu C. w. Hsu C. Zhou S. Schwartz D. C. Bayles D. O.. (2012). Genome sequencing of ovine isolates of *Mycobacterium avium* subspecies *paratuberculosis* offers insights into host association. BMC Genomics 13, 89. 10.1186/1471-2164-13-89 22409516 PMC3337245

[B5] Barrangou R. Fremaux C. Deveau H. Richards M. Boyaval P. Moineau S.. (2007). Against viruses in prokaryotes. Science 315, 1709–1712. 10.1126/science.1138140 17379808

[B6] Beloglazova N. Petit P. Flick R. Brown G. Savchenko A. Yakunin A. F.. (2011). Structure and activity of the Cas3 HD nuclease MJ0384, an effector enzyme of the CRISPR interference. EMBO J. 30, 4616–4627. 10.1038/emboj.2011.377 22009198 PMC3243599

[B7] Blouin Y. Cazajous G. Dehan C. Soler C. Vong R. Hassan M. O.. (2014). Progenitor “*Mycobacterium canettii*” Clone responsible for lymph node tuberculosis epidemic, Djibouti. Emerg. Infect. Dis. 20, 21–28. 10.3201/eid2001.130652 24520560 PMC3884719

[B8] Boritsch E. C. Khanna V. Pawlik A. Honoré N. Navas V. H. Ma L.. (2016). Key experimental evidence of chromosomal DNA transfer among selected tuberculosis causing mycobacteria. Proc. Natl. Acad. Sci. U. S. A. 113, 9876–9881. 10.1073/pnas.1604921113 27528665 PMC5024641

[B9] Boritsch E. C. Supply P. Honoré N. Seeman T. Stinear T. P. Brosch R.. (2014). A glimpse into the past and predictions for the future: the molecular evolution of the tuberculosis agent. Mol. Microbiol. 93, 835–852. 10.1111/mmi.12720 25039682

[B10] Brendel J. Stoll B. Lange S. J. Sharma K. Lenz C. Stachler A. E.. (2014). A complex of cas proteins 5, 6, and 7 is required for the biogenesis and stability of clustered regularly interspaced short palindromic repeats (CRISPR)-derived RNAs (crRNAs) in haloferax volcanii. J. Biol. Chem. 289, 7164–7177. 10.1074/jbc.M113.508184 24459147 PMC3945376

[B11] Brettin T. Davis J. J. Disz T. Edwards R. A. Gerdes S. Olsen G. J.. (2015). RASTtk: a modular and extensible implementation of the RAST algorithm for building custom annotation pipelines and annotating batches of genomes. Sci. Rep. 5, 8365. 10.1038/srep08365 25666585 PMC4322359

[B12] Camacho C. Coulouris G. Avagyan V. Ma N. Papadopoulos J. Bealer K.. (2009). BLAST+: architecture and applications. BMC Bioinformatics 10, 1–9. 10.1186/1471-2105-10-421 20003500 PMC2803857

[B13] Coitinho C. Greif G. van Ingen J. Laserra P. Robello C. Rivas C.. (2016). First case of *Mycobacterium heckeshornense* cavitary lung disease in the Latin America and Caribbean region. New Microbes New Infect. 9, 63–65. 10.1016/j.nmni.2015.12.003 26909156 PMC4735480

[B14] Couvin D. Bernheim A. Toffano-Nioche C. Touchon M. Michalik J. Néron B.. (2018). CRISPRCasFinder, an update of CRISRFinder, includes a portable version, enhanced performance and integrates search for Cas proteins. Nucleic Acids Res. 46, W246–W251. 10.1093/nar/gky425 29790974 PMC6030898

[B15] Darriba D. i. Posada D. Kozlov A. M. Stamatakis A. Morel B. Flouri T.. (2020). ModelTest-NG: a new and scalable tool for the selection of DNA and protein evolutionary models. Mol. Biol. Evol. 37, 291–294. 10.1093/molbev/msz189 31432070 PMC6984357

[B16] Derbyshire K. M. Gray T. A. (2014). Distributive conjugal transfer: new insights into horizontal gene transfer and genetic exchange in mycobacteria. Microbiol. Spectr. 2, 1–32. 10.1128/microbiolspec.MGM2-0022-2013 25505644 PMC4259119

[B17] Dumas E. Boritsch E. C. Vandenbogaert M. De La Vega R. C. R. Thiberge J. M. Caro V.. (2016). Mycobacterial pan-genome analysis suggests important role of plasmids in the radiation of type VII secretion systems. Genome Biol. Evol. 8, 387–402. 10.1093/gbe/evw001 26748339 PMC4779608

[B18] Edgar R. C. (2004). MUSCLE: multiple sequence alignment with high accuracy and high throughput. Nucleic Acids Res. 32, 1792–1797. 10.1093/nar/gkh340 15034147 PMC390337

[B19] Garneau J. E. Dupuis M.-È. Villion M. Romero D. A. Barrangou R. Boyaval P.. (2010). The CRISPR/Cas bacterial immune system cleaves bacteriophage and plasmid DNA. Nature 468, 67–71. 10.1038/nature09523 21048762

[B20] Garside E. L. Schellenberg M. J. Gesner E. M. Bonanno J. B. Sauder J. M. Burley S. K.. (2012). Cas5d processes pre-crRNA and is a member of a larger family of CRISPR RNA endonucleases. RNA 18, 2020–2028. 10.1261/rna.033100.112 23006625 PMC3479392

[B21] Gasteiger E. Gattiker A. Hoogland C. Ivanyi I. Appel R. D. Bairoch A.. (2003). ExPASy: the proteomics server for in-depth protein knowledge and analysis. Nucleic Acids Res. 31, 3784–3788. 10.1093/nar/gkg563 12824418 PMC168970

[B22] Gray T. Derbyshire K. (2018). Blending genomes: distributive conjugal transfer in mycobacteria, a sexier form of HGT. Mol. Microbiol. 108, 601–613. 10.1111/mmi.13971 29669186 PMC5997560

[B23] Groenen P. M. A. Bunschoten A. E. van Soolingen D. Errtbden J. D. A. (1993). Nature of DNA polymorphism in the direct repeat cluster of *Mycobacterium tuberculosis*; application for strain differentiation by a novel typing method. Mol. Microbiol. 10, 1057–1065. 10.1111/j.1365-2958.1993.tb00976.x 7934856

[B24] Grüschow S. Athukoralage J. S. Graham S. Hoogeboom T. White M. F. (2019). Cyclic oligoadenylate signalling mediates *Mycobacterium tuberculosis* CRISPR defence. Nucleic Acids Res. 47, 9259–9270. 10.1093/nar/gkz676 31392987 PMC6755085

[B25] Guan Q. Garbati M. Mfarrej S. Almutairi T. Laval T. Singh A.. (2021). Insights into the ancestry evolution of the *Mycobacterium tuberculosis* complex from analysis of *Mycobacterium riyadhense*. NAR Genom. Bioinform. 3, 1–16. 10.1093/nargab/lqab070 34396095 PMC8356964

[B26] He L. Fan X. Xie J. (2012). Comparative genomic structures of *Mycobacterium* CRISPR-Cas. J. Cell Biochem. 113, 2464–2473. 10.1002/jcb.24121 22396173

[B27] Hochstrasser M. L. Taylor D. W. Kornfeld J. E. Nogales E. Doudna J. A. (2016). DNA Targeting by a minimal CRISPR RNA-guided cascade. Mol. Cell 63, 840–851. 10.1016/j.molcel.2016.07.027 27588603 PMC5111854

[B28] Horan K. L. Freeman R. Weigel K. Semret M. Pfaller S. Covert T. C.. (2006). Isolation of the genome sequence strain *Mycobacterium avium* 104 from multiple patients over a 17-year period. J. Clin. Microbiol. 44, 783–789. 10.1128/JCM.44.3.783-789.2006 16517855 PMC1393153

[B29] Hrle A. Su A. A. H. Ebert J. Benda C. Randau L. Conti E.. (2013). Structure and RNA-binding properties of the type III-A CRISPR-associated protein Csm3. RNA Biol. 10, 1670–1678. 10.4161/rna.26500 24157656 PMC3907477

[B30] Iitoh E. Tominaga M. Okamoto M. Sakazaki Y. Nakamura M. Kinoshita T.. (2020). A case of pulmonary *Mycobacterium heckeshornense* infection in a healthy Japanese man: a case of pulmonary *M. heckeshornense* infection. Respir. Med. Case Rep. 30, 101093. 10.1016/j.rmcr.2020.101093 32489849 PMC7256317

[B31] Jagielski T. Borówka P. Bakuła Z. Lach J. Marciniak B. Brzostek A.. (2020). Genomic insights into the *Mycobacterium kansasii* complex: an update. Front. Microbiol. 10, 2918. 10.3389/fmicb.2019.02918 32010067 PMC6974680

[B32] Judd J. A. Canestrari J. Clark R. Joseph A. Lapierre P. Lasek-nesselquist E.. (2021). A mycobacterial systems resource for the research community. MBio 12, 1–15. 10.1128/mBio.02401-20 33653882 PMC8092266

[B33] Kamerbeek J. Schouls L. Kolk A. Van Agterveld M. Van Soolingen D. Kuijper S.. (1997). Simultaneous detection and strain differentiation of *Mycobacterium tuberculosis* for diagnosis and epidemiology. J. Clin. Microbiol. 35, 907–914. 10.1128/jcm.35.4.907-914.1997 9157152 PMC229700

[B34] Korea Institute of Geoscience Mineral Resources (2021). Mycobacterium sp. SM1. NCBI. Available online at: https://www.ncbi.nlm.nih.gov/data-hub/genome/GCA_018361265.1./ (accessed April 3, 2023).

[B35] Kozlov A. M. Darriba D. Flouri T. Morel B. Stamatakis A. (2019). RAxML-NG: A fast, scalable and user-friendly tool for maximum likelihood phylogenetic inference. Bioinformatics 35, 4453–4455. 10.1093/bioinformatics/btz305 31070718 PMC6821337

[B36] Lundgren M. Charpentier E. Fineran P. C. (2015). CRISPR: methods and protocols. Cris. Methods Protoc. 1311, 1–366. 10.1007/978-1-4939-2687-9

[B37] Madacki J. Orgeur M. Fiol G. M. Frigui W. Ma L. Brosch R.. (2021). Esx-1-independent horizontal gene transfer by *Mycobacterium tuberculosis* complex strains. MBio 12, e00965-21. 10.1128/mBio.00965-21 34006663 PMC8262963

[B38] Makarova K. S. Wolf Y. I. Iranzo J. Shmakov S. A. Alkhnbashi O. S. Brouns S. J. J.. (2020). Evolutionary classification of CRISPR–Cas systems: a burst of class 2 and derived variants. Nat. Rev. Microbiol. 18, 67–83. 10.1038/s41579-019-0299-x 31857715 PMC8905525

[B39] Marri P. R. Bannantine J. P. Golding G. B. (2006). Comparative genomics of metabolic pathways in *Mycobacterium* species: gene duplication, gene decay and lateral gene transfer. FEMS Microbiol. Rev. 30, 906–925. 10.1111/j.1574-6976.2006.00041.x 17064286

[B40] Olson R. D. Assaf R. Brettin T. Conrad N. Cucinell C. Davis J. J.. (2022). Introducing the bacterial and viral bioinformatics resource center (BV-BRC): a resource combining PATRIC, IRD and ViPR. Nucleic Acids Res. 51, 678–689. 10.1093/nar/gkac1003 36350631 PMC9825582

[B41] Otal I. Martin C. Vincent-Levy-Frebault V. Thierry D. Gicquel B. (1991). Restriction fragment length polymorphism analysis using IS6110 as an epidemiological marker in tuberculosis. J Clin. Microbiol. 29, 1252–1254. 10.1128/jcm.29.6.1252-1254.1991 1677943 PMC269979

[B42] Panda A. Drancourt M. Tuller T. Pontarotti P. (2018). Genome-wide analysis of horizontally acquired genes in the genus *Mycobacterium*. Sci. Rep. 8, 1–13. 10.1038/s41598-018-33261-w 30287860 PMC6172269

[B43] Pinilla-Redondo R. Mayo-Muñoz D. Russel J. Garrett R. A. Randau L. Sørensen S. J.. (2020). Type IV CRISPR-Cas systems are highly diverse and involved in competition between plasmids. Nucleic Acids Res. 48, 2000–2012. 10.1093/nar/gkz1197 31879772 PMC7038947

[B44] Pinilla-Redondo R. Russel J. Mayo-Muñoz D. Shah S. A. Garrett R. A. Nesme J.. (2022). CRISPR-Cas systems are widespread accessory elements across bacterial and archaeal plasmids. Nucleic Acids Res. 50, 4315–4328. 10.1093/nar/gkab859 34606604 PMC9071438

[B45] Samai P. Smith P. Shuman S. (2010). Structure of a CRISPR-associated protein Cas2 from *Desulfovibrio vulgaris*. Acta Crystallogr. Sect. F Struct. Biol. Cryst. Commun. 66, 1552–1556. 10.1107/S1744309110039801 21139194 PMC2998353

[B46] Sanchini A. Semmler T. Mao L. Kumar N. Dematheis F. Tandon K.. (2016). A hypervariable genomic island identified in clinical and environmental *Mycobacterium avium* subsp. *hominissuis* isolates from Germany. Int. J. Med. Microbiol. 306, 495–503. 10.1016/j.ijmm.2016.07.001 27481640

[B47] Sapriel G. Brosch R. Bapteste E. (2019). Shared pathogenomic patterns characterize a new phylotype, revealing transition toward host-adaptation long before speciation of *Mycobacterium tuberculosis*. Genome Biol. Evol. 11, 2420–2438. 10.1093/gbe/evz162 31368488 PMC6736058

[B48] Sayers E. W. Bolton E. E. Brister J. R. Canese K. Chan J. Comeau D. C.. (2022). Database resources of the national center for biotechnology information. Nucleic Acids Res. 50, D20–D26. 10.1093/nar/gkab1112 34850941 PMC8728269

[B49] Semret M. Zhai G. Mostowy S. Cleto C. Alexander D. Cangelosi G.. (2004). Extensive genomic polymorphism within *Mycobacterium avium*. J. Bacteriol. 186, 6332–6334. 10.1128/JB.186.18.6332-6334.2004 15342607 PMC515132

[B50] Shahraki A. H. Trovato A. Mirsaeidi M. Borroni E. Heidarieh P. Hashemzadeh M.. (2017). Mycobacterium persicum sp. Nov., a novel species closely related to *Mycobacterium kansasii* and *Mycobacterium gastri. Int. J. Syst. Evol. Microbiol*. 67, 1758–1765. 10.1099/ijsem.0.001862 28629501

[B51] Shangguan Q. Graham S. Sundaramoorthy R. White M. F. (2022). Structure and mechanism of the type I-G CRISPR effector. Nucleic Acids Res. 50, 11214–11228. 10.1093/nar/gkac925 36305833 PMC9638904

[B52] Singh A. Gaur M. Sharma V. Khanna P. Bothra A. Bhaduri A.. (2021). Comparative genomic analysis of *Mycobacteriaceae* reveals horizontal gene transfer-mediated evolution of the CRISPR-Cas system in the *Mycobacterium tuberculosis* complex. mSystems 6, e00934-20. 10.1128/mSystems.00934-20 33468705 PMC7820667

[B53] Sreevatsan S. Pan X. Stockbauer K. E. Connell N. D. Kreiswirth B. N. Whittam T. S.. (1997). Restricted structural gene polymorphism in the *Mycobacterium tuberculosis* complex indicates evolutionarily recent global dissemination. Proc. Natl. Acad. Sci. U. S. A. 94, 9869–9874. 10.1073/pnas.94.18.9869 9275218 PMC23284

[B54] Supply P. Marceau M. Mangenot S. Roche D. (2013). Genome analysis of smooth tubercle bacilli provides insights into ancestry and pathoadaptation of the etiologic agent of tuberculosis. Nat. Genet. 45, 172–179. 10.1038/ng.2517 23291586 PMC3856870

[B55] Tagini F. Aeby S. Bertelli C. Droz S. Casanova C. Prod'Hom G.. (2019). Phylogenomics reveal that *Mycobacterium kansasii* subtypes are species-level lineages. Description of *Mycobacterium pseudokansasii* sp. nov*., Mycobacterium innocens* sp. nov. and *Mycobacterium attenuatum* sp. nov. Int. J. Syst. Evol. Microbiol. 69, 1696–1704. 10.1099/ijsem.0.003378 30950782

[B56] Tagini F. Pillonel T. Bertelli C. Jaton K. Greub G. (2021). Pathogenic determinants of the *Mycobacterium kansasii* complex: an unsuspected role for distributive conjugal transfer. Microorganisms 9, 1–22. 10.3390/microorganisms9020348 33578772 PMC7916490

[B57] Tatusova T. Dicuccio M. Badretdin A. Chetvernin V. Nawrocki E. P. Zaslavsky L.. (2016). NCBI prokaryotic genome annotation pipeline. Nucleic Acids Res. 44, 6614–6624. 10.1093/nar/gkw569 27342282 PMC5001611

[B58] Ummels R. Abdallah A. M. Kuiper V. Aâjoud A. Sparrius M. Naeem R.. (2014). Identification of a novel conjugative plasmid in mycobacteria that requires both type IV and type VII secretion. MBio 5, 1–8. 10.1128/mBio.01744-14 25249284 PMC4173767

[B59] Van Hest R. Van Der Zanden A. Boeree M. Kremer K. Dessens M. Westenend P.. (2004). *Mycobacterium heckeshornense* infection in an immunocompetent patient and identification by 16S rRNA sequence analysis of culture material and a histopathology tissue specimen. J. Clin. Microbiol. 42, 4386–4389. 10.1128/JCM.42.9.4386-4389.2004 15365051 PMC516325

[B60] van Ingen J. Al-Haijoj S. A. M. Boeree M. Al-Rabiah F. Enaimi M. de Zwaan R.. (2009). *Mycobacterium riyadhense* sp. nov., a non-tuberculous species identified as *Mycobacterium tuberculosis* complex by a commercial line-probe assay. Int. J. Syst. Evol. Microbiol. 59, 1049–1053. 10.1099/ijs.0.005629-0 19406791

[B61] Veyrier F. Pletzer D. Turenne C. Behr M. A. (2009). Phylogenetic detection of horizontal gene transfer during the step-wise genesis of *Mycobacterium tuberculosis*. BMC Evol. Biol. 9, 1–14. 10.1186/1471-2148-9-196 19664275 PMC3087520

[B62] Vissa V. D. Brennan P. J. (2001). The genome of *Mycobacterium leprae*: a minimal mycobacterial gene set. Genome Biol. 2, 1–8. 10.1186/gb-2001-2-8-reviews1023 11532219 PMC138955

[B63] Wei W. Zhang S. Fleming J. Chen Y. Li Z. Fan S.. (2019). *Mycobacterium tuberculosis* type III-A CRISPR/Cas system crRNA and its maturation have atypical features. FASEB J. 33, 1496–1509. 10.1096/fj.201800557RR 29979631

[B64] Wheatley R. M. MacLean R. C. (2021). CRISPR-Cas systems restrict horizontal gene transfer in *Pseudomonas aeruginosa*. ISME J. 15, 1420–1433. 10.1038/s41396-020-00860-3 33349652 PMC8105352

[B65] Zhang J. Kasciukovic T. White M. F. (2012). The CRISPR associated protein Cas4 Is a 5′ to 3′ DNA exonuclease with an iron-sulfur cluster. PLoS ONE 7, e0047232. 10.1371/journal.pone.0047232 23056615 PMC3466216

[B66] Zhang Q. Ye Y. (2017). Not all predicted CRISPR-Cas systems are equal: Isolated cas genes and classes of CRISPR like elements. BMC Bioinformatics 18, 1–12. 10.1186/s12859-017-1512-4 28166719 PMC5294841

