# Supplementary material for: Cold Cas: reevaluating the occurrence of CRISPR/Cas systems in Mycobacteriaceae
Source: Front Microbiol. 2023 Jun 27;14:1204838. doi: 10.3389/fmicb.2023.1204838 (PMC10333696; doi:10.3389/fmicb.2023.1204838)
Supplement: Supplementary file 1 [file Presentation_1.zip › Data Sheet 3.pdf]

Physical cores: 32

Logical cores: 64

Memory: 251GB

Extensions: AVX

WARNING: Partition DATA: Sequences Mycobacterium_canettii_CIPT_140070005_STB-G_Locus and Mycobacterium_canettii_CIPT-140070007_STB-I_Locus are identical

WARNING: Partition DATA: Sequences Mycobacterium_canettii_CIPT_140010059 and Mycobacterium_tuberculosis_1.1.2_Locus are identical

WARNING: Partition DATA: Sequences Mycobacterium_canettii_CIPT_140010059 and Mycobacterium_tuberculosis_3.1.1_Locus are identical

WARNING: Partition DATA: Sequences Mycobacterium_canettii_CIPT_140010059 and Mycobacterium_tuberculosis_4.1_Locus are identical

WARNING: Partition DATA: Sequences Mycobacterium_canettii_CIPT_140010059 and Mycobacterium_bovis_AF2122-97_Locus are identical

WARNING: Partition DATA: Sequences Mycobacterium_canettii_CIPT_140010059 and Mycobacterium_africanum_str._25_Locus are identical

WARNING: Partition DATA: Sequences Mycobacterium_canettii_CIPT_140010059 and Mycobacterium_canettii_Percy32_Locus are identical

WARNING: Partition DATA: Sequences Mycobacterium_canettii_CIPT_140010059 and Mycobacterium_canettii_CIPT-140060008_Locus1 are identical

WARNING: Partition DATA: Sequences Mycobacterium_innocens_49_11_Locus1 and Mycobacterium_innocens_MK13_Locus1 are identical

WARNING: Partition DATA: Sequences Mycobacterium_innocens_49_11_Locus2 and Mycobacterium_innocens_MK13_Locus2 are identical

WARNING: Partition DATA: Sequences Mycobacterium_ostraviense_1010001458_Locus and Mycobacterium_ostraviense_241-15_Locus are identical

WARNING: Partition DATA: Sequences Mycobacterium_riyadhense_MR-246_Locus and Mycobacterium_riyadhense_MR-1023_Locus are identical

WARNING: Partition DATA: Sequences Mycobacterium_canettii_CIPT_140070013_STB-H_Locus and Mycobacterium_canettii_CIPT-140070002_STB-E_Locus are identical

WARNING: Partition DATA: Sequences Mycobacterium_canettii_CIPT_140070013_STB-H_Locus and Mycobacterium_canettii_NLA000701671_Locus are identical

WARNING: Partition DATA: Sequences Mycobacterium_canettii_CIPT_140070013_STB-H_Locus and Mycobacterium_canettii_Percy525_Locus are identical

WARNING: Partition DATA: Sequences Mycobacterium_canettii_CIPT_140070013_STB-H_Locus and Mycobacterium_canettii_CIPT-140060008_Locus2 are identical

WARNING: MSA has not enough sites to infer reliable results

Creating new checkpoint file: Cas1MSA_ModelTest.txt.ckp

ModelTest-NG v0.1.7

Input data:

MSA: Cas1MSA.aln

Tree: Maximum parsimony

file: -

#taxa: 42

#sites: 585

#patterns: 571

Max. thread mem: 56 MB

Output:

Log: Cas1MSA_ModelTest.txt.log

Starting tree: Cas1MSA_ModelTest.txt.tree

Results: Cas1MSA_ModelTest.txt.out

Selection options:

protein matrices: 19

protein models: 152

include model parameters:

Uniform: true

p-inv (+I): true

gamma (+G): true

both (+I+G): true

free rates (+R): false

fixed freqs: true

estimated freqs: false

#categories: 4

gamma rates mode: mean

asc bias: none

epsilon (opt): 0.01

epsilon (par): 0.05

keep branches: false

Additional options:

verbosity: very low

threads: 60/32

RNG seed: 12345

subtree repeats: enabled

----

modeltest-ng was called as follows:

>> modeltest-ng -i Cas1MSA.aln -d aa -p 60 -o Cas1MSA_ModelTest.txt

Partition 1/1

----ID--- ----MODEL---- ---Time--- -Elapsed--- -------LnL------- -Alpha- -P-inv-

Computation of likelihood scores completed. It took 0h:00:02

BIC model K lnL score delta weight

1	LG+G4+F	20	-11473.2580	23590.0487	0.0000	0.9816
2	LG+I+G4+F	21	-11474.0506	23598.0057	7.9570	0.0184
3	WAG+G4+F	20	-11514.0322	23671.5973	81.5486	0.0000
4	WAG+I+G4+F	21	-11512.3316	23674.5676	84.5189	0.0000
5	RTREV+G4+F	20	-11520.7845	23685.1019	95.0532	0.0000
6	RTREV+I+G4+F	21	-11521.2056	23692.3156	102.2668	0.0000
7	VT+G4+F	20	-11537.1295	23717.7918	127.7431	0.0000
8	VT+I+G4+F	21	-11537.5882	23725.0809	135.0322	0.0000
9	JTT+G4+F	20	-11560.7293	23764.9913	174.9426	0.0000
10	JTT-DCMUT+G4+F	20	-11562.3847	23768.3022	178.2535	0.0000

Best model according to BIC

Model: LG+G4+F

lnL: -11473.2580

Frequencies: 0.1056 0.1009 0.0230 0.0608 0.0140 0.0397 0.0535 0.0701 0.0207 0.0451 0.0999 0.0218 0.0173 0.0339 0.0485 0.0635 0.0532 0.0098 0.0295 0.0894

Inv. sites prop: -

Gamma shape: 1.5901

Score: 23590.0487

Weight: 0.9816

Parameter importances

P.Inv: 0.0000

Gamma: 0.9816

Gamma-Inv: 0.0184

Frequencies: 1.0000

Model averaged estimates

P.Inv: 0.0231

Alpha: 1.5901

Alpha-P.Inv: 1.7485

P.Inv-Alpha: 0.0221

Frequencies: 0.1056 0.1009 0.0230 0.0608 0.0140 0.0397 0.0535 0.0701 0.0207 0.0451 0.0999 0.0218 0.0173 0.0339 0.0485 0.0635 0.0532 0.0098 0.0295 0.0894

Commands:

```
 > phyml  -i Cas1MSA.aln -d aa -m LG -f e -v 0 -a e -c 4 -o tlr

 > raxmlHPC-SSE3 -s Cas1MSA.aln -m PROTGAMMALGF -n EXEC_NAME -p PARSIMONY_SEED

 > raxml-ng --msa Cas1MSA.aln --model LG+G4+F

 > paup -s Cas1MSA.aln

 > iqtree -s Cas1MSA.aln -m LG+G4+F
```

AIC	model	K	lnL	score	delta	weight
1	LG+G4+F	20	-11473.2580	23148.5159	0.0000	0.8573
2	LG+I+G4+F	21	-11474.0506	23152.1013	3.5854	0.1427
3	WAG+I+G4+F	21	-11512.3316	23228.6632	80.1472	0.0000
4	WAG+G4+F	20	-11514.0322	23230.0645	81.5486	0.0000
5	RTREV+G4+F	20	-11520.7845	23243.5691	95.0532	0.0000
6	RTREV+I+G4+F	21	-11521.2056	23246.4112	97.8952	0.0000
7	VT+G4+F	20	-11537.1295	23276.2590	127.7431	0.0000
8	VT+I+G4+F	21	-11537.5882	23279.1765	130.6606	0.0000
9	JTT+G4+F	20	-11560.7293	23323.4585	174.9426	0.0000
10	JTT-DCMUT+G4+F	20	-11562.3847	23326.7694	178.2535	0.0000

Best model according to AIC

Model: LG+G4+F

lnL: -11473.2580

Frequencies: 0.1056 0.1009 0.0230 0.0608 0.0140 0.0397 0.0535 0.0701 0.0207 0.0451 0.0999 0.0218 0.0173 0.0339 0.0485 0.0635 0.0532 0.0098 0.0295 0.0894

Inv. sites prop: -

Gamma shape: 1.5901

Score: 23148.5159

Weight: 0.8573

Parameter importances

P.Inv: 0.0000

Gamma: 0.8573

Gamma-Inv: 0.1427

Frequencies: 1.0000

Model averaged estimates

P.Inv: 0.0231

Alpha: 1.5901

Alpha-P.Inv: 1.7485

P.Inv-Alpha: 0.0221

Frequencies: 0.1056 0.1009 0.0230 0.0608 0.0140 0.0397 0.0535 0.0701 0.0207 0.0451 0.0999 0.0218 0.0173 0.0339 0.0485 0.0635 0.0532 0.0098 0.0295 0.0894

Commands:

```
> phyml  -i Cas1MSA.aln -d aa -m LG -f e -v 0 -a e -c 4 -o tlr

> raxmlHPC-SSE3 -s Cas1MSA.aln -m PROTGAMMALGF -n EXEC_NAME -p PARSIMONY_SEED

> raxml-ng --msa Cas1MSA.aln --model LG+G4+F

> paup -s Cas1MSA.aln

> iqtree -s Cas1MSA.aln -m LG+G4+F
```

```
AICc     model            K        lnL          score         delta    weight
--------------------------------------------------------------------------------
      1  LG+G4+F          20    -11473.2580    23190.5159       0.0000    0.9083
      2  LG+I+G4+F        21    -11474.0506    23195.1013       4.5854    0.0917
      3  WAG+I+G4+F       21    -11512.3316    23271.6632      81.1472    0.0000
      4  WAG+G4+F         20    -11514.0322    23272.0645      81.5486    0.0000
      5  RTREV+G4+F       20    -11520.7845    23285.5691      95.0532    0.0000
      6  RTREV+I+G4+F     21    -11521.2056    23289.4112      98.8952    0.0000
      7  VT+G4+F          20    -11537.1295    23318.2590     127.7431    0.0000
      8  VT+I+G4+F        21    -11537.5882    23322.1765     131.6606    0.0000
      9  JTT+G4+F         20    -11560.7293    23365.4585     174.9426    0.0000
     10  JTT-DCMUT+G4+F   20    -11562.3847    23368.7694     178.2535    0.0000
--------------------------------------------------------------------------------
Best model according to AICc
---------------------------
Model:            LG+G4+F
lnL:              -11473.2580
Frequencies:      0.1056 0.1009 0.0230 0.0608 0.0140 0.0397 0.0535 0.0701 0.0207 0.0451 0.0999
0.0218 0.0173 0.0339 0.0485 0.0635 0.0532 0.0098 0.0295 0.0894
Inv. sites prop:  -
Gamma shape:      1.5901
Score:            23190.5159
Weight:           0.9083
```

Parameter importances

P.Inv: 0.0000

Gamma: 0.9083

Gamma-Inv: 0.0917

Frequencies: 1.0000

Model averaged estimates

P.Inv: 0.0231

Alpha: 1.5901

Alpha-P.Inv: 1.7485

P.Inv-Alpha: 0.0221

Frequencies: 0.1056 0.1009 0.0230 0.0608 0.0140 0.0397 0.0535 0.0701 0.0207 0.0451 0.0999 0.0218 0.0173 0.0339 0.0485 0.0635 0.0532 0.0098 0.0295 0.0894

Commands:

```
> phyml  -i Cas1MSA.aln -d aa -m LG -f e -v 0 -a e -c 4 -o tlr
> raxmlHPC-SSE3 -s Cas1MSA.aln -m PROTGAMMALGF -n EXEC_NAME -p PARSIMONY_SEED
> raxml-ng --msa Cas1MSA.aln --model LG+G4+F
> paup -s Cas1MSA.aln
> iqtree -s Cas1MSA.aln -m LG+G4+F
```

Summary:

Partition 1/1:

	Model	Score	Weight
BIC	LG+G4+F	23590.0487	0.9816

AIC	LG+G4+F	23148.5159	0.8573
AICc	LG+G4+F	23190.5159	0.9083

Execution results written to Cas1MSA_ModelTest.txt.out

Starting tree written to Cas1MSA_ModelTest.txt.tree
