# Supplementary material for: Cold Cas: reevaluating the occurrence of CRISPR/Cas systems in Mycobacteriaceae
Source: Front Microbiol. 2023 Jun 27;14:1204838. doi: 10.3389/fmicb.2023.1204838 (PMC10333696; doi:10.3389/fmicb.2023.1204838)
Supplement: Supplementary file 1 [file Presentation_1.zip › List of Supplementary Files.docx]

Supplementary Material

Cold Cas: Reevaluating rarity of CRISPR/Cas systems across *Mycobacteriaceae*

Evan Brenner^1^, Srinand Sreevatsan^1*^

*** Correspondence:** Srinand Sreevatsan: sreevats@msu.edu

# Supplementary Data

**Supplemental File 1:** Supplemental1_ConcatenatedMycobacterialCas1.fasta

A total of 42 FASTA-formatted mycobacterial Cas1 protein sequences. This is not a complete set of mycobacterial Cas systems and is only representative.

**Supplemental File 2:** Supplemental2_CasAlignment.fasta

FASTA output of MUSCLE v3.8.31 as described in Methods, for alignment of mycobacterial Cas protein sequences in Supplemental File 1.

**Supplemental File 3:** Supplemental3_ModelTestOutput.pdf

The terminal output for ModelTest-NG for the best substitution model to use for phylogenetic reconstruction through RAxML-NG used in Figure 5. ModelTest-NG assesses best models by three ranking systems: Akaike Information Criterion (AIC), Bayesian Information Criterion (BIC), and Akaike Information Criterion, Corrected (AIC_C_). In all cases, the substitution model VT+G4+F was selected as the best performing model, and was used for RAxML-NG phylogenetic reconstruction subsequently.

**Supplemental File 4:** Supplemental4_PhylogeneticTreeFiles.zip

ZIP archive containing the raw phylogenetic tree data. These unmodified data are provided for full transparency, as the tree presented in Figure 5 was first visualized in FigTree software and edited for visual clarity by Inkscape software:

CasTree_BestMLTree.fasta.raxml.bestTree: Best scoring maximum likelihood tree as identified by RAxML-NG by parameters described in Methods. File is in Newick format.

CasTree_Bootstraps_AutoMRE.fasta.raxml.bootstraps: Set of 1000 bootstrap replicates as performed by RAxML-NG by parameters described in Methods. File is in Newick format.

CasTree_WithBootstrapSupport.nwk: Best scoring maximum likelihood tree with bootstrap support values appended to the nodes by RAxML-NG as described in Methods. File is in Newick format.
