# Supplementary figures and images for: Cold Cas: reevaluating the occurrence of CRISPR/Cas systems in Mycobacteriaceae
Source: Front Microbiol. 2023 Jun 27;14:1204838. doi: 10.3389/fmicb.2023.1204838 (PMC10333696; doi:10.3389/fmicb.2023.1204838)

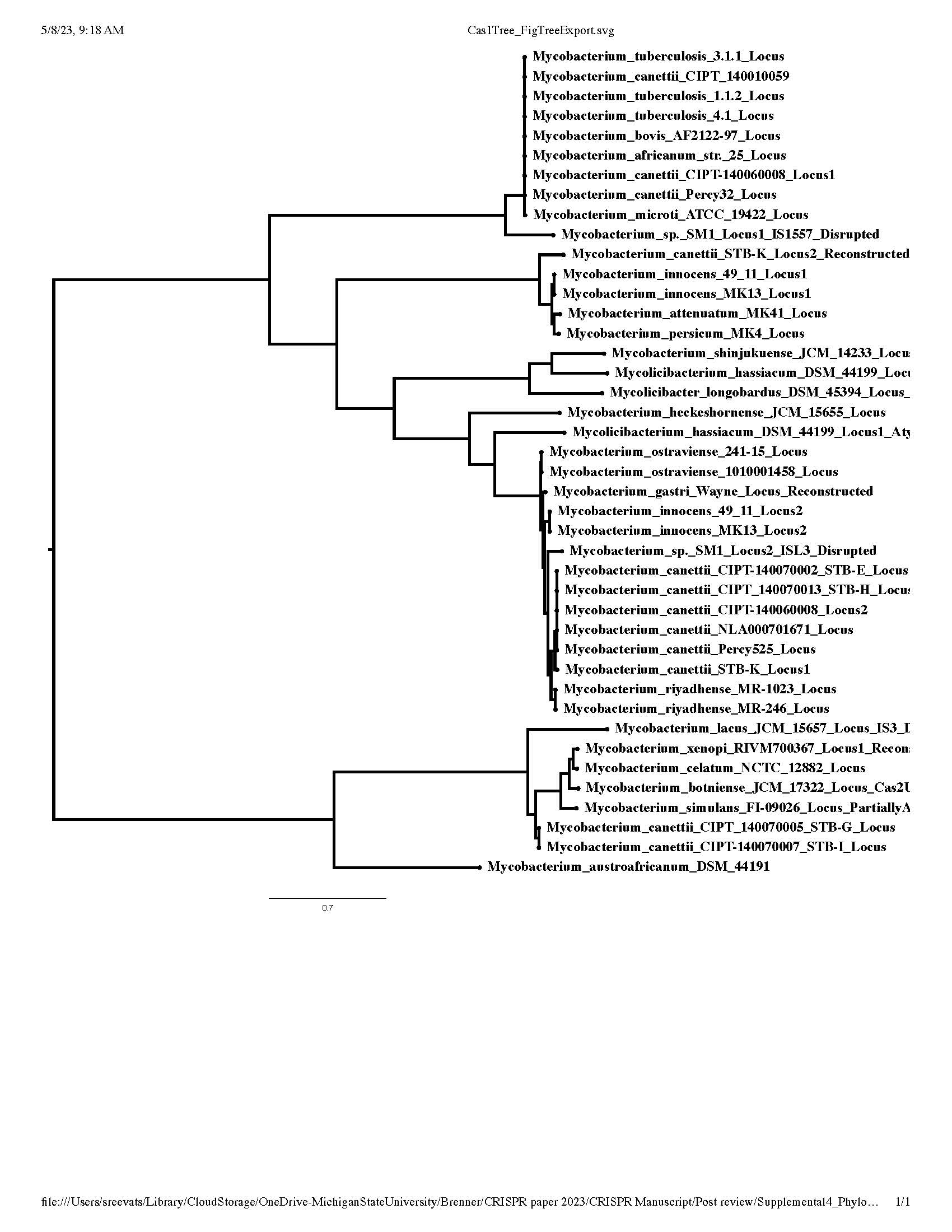

Supplement: Supplementary file 1 [file Presentation_1.zip › Image 1.JPEG]
